# The clinical course of SARS-CoV-2 infection in patients with glomerular diseases and evaluation of the subsequent risk of relapse

**DOI:** 10.3389/fneph.2024.1472294

**Published:** 2024-10-21

**Authors:** Sophia Lionaki, Evangelia Dounousi, Smaragdi Marinaki, Konstantia Kantartzi, Marios Papasotiriou, Dimitra Galitsiou, Ioannis Bellos, Aggeliki Sardeli, Petros Kalogeropoulos, Vassilios Liakopoulos, Christos Mpintas, Dimitrios Goumenos, Sophia Flouda, Aliki Venetsanopoulou, Paraskevi Voulgari, Eva Andronikidi, Georgios Moustakas, Stylianos Panagoutsos, Ioannis Boletis

**Affiliations:** ^1^ Division of Nephrology, 2nd Department of Propaedeutic Internal Medicine, Attikon University Hospital, School of Medicine, National and Kapodistrian University of Athens, Athens, Greece; ^2^ Department of Nephrology, University Hospital of Ioannina, Faculty of Medicine, University of Ioannina, Ioannina, Greece; ^3^ Clinic of Nephrology and Renal Transplantation, Laiko General Hospital, School of Health Sciences, National and Kapodistrian University of Athens, Athens, Greece; ^4^ Department of Nephrology, School of Health Sciences, Democritus University of Thrace, Alexandroupolis, Greece; ^5^ Department of Nephrology and Renal Transplantation, University Hospital of Patras, Patras, Greece; ^6^ Department of Nephrology, Peripheral General Hospital Athens Giorgos Gennimatas, Athens, Greece; ^7^ 2nd Department of Nephrology, AHEPA University Hospital, Medical school, Aristotle University of Thessaloniki, Thessaloniki, Greece; ^8^ 4th Department of Internal Medicine, Attikon University Hospital, School of Medicine, National and Kapodistrian University of Athens, Athens, Greece; ^9^ Department of Rheumatology, University Hospital of Ioannina, Faculty of Medicine, University of Ioannina, Ioannina, Greece; ^10^ Department of Nephrology, Aretaieio Hospital, School of Medicine, National and Kapodistrian University of Athens, Athens, Greece

**Keywords:** COVID-19, glomerulonephritis, outcome, podocytopathies, relapse

## Abstract

**Introduction:**

This study aimed to describe the clinical course of severe acute respiratory syndrome coronavirus 2 (SARS-CoV-2) infection in patients with glomerular diseases (GDs) and its impact on the probability of relapse.

**Methods:**

Patients with biopsy-proven GD and positive PCR test for SARS-CoV-2 from glomerular clinics across Greece were studied retrospectively. Those who received the GD diagnosis after the SARS-CoV-2 vaccination or coronavirus disease 2019 (COVID-19) or ended in ESKD prior to infection were excluded. Demographics, histopathological diagnoses, past medical history, immunosuppression, and GD activity status were recorded.

**Results:**

A total of 219 patients with GDs and documented SARS-CoV-2 infection were included. The mean time from the diagnostic kidney biopsy to SARS-CoV-2 infection was 67.6 ( ± 59.3) months. Among the participants, 82.5% had been vaccinated against SARS-CoV-2 with three doses (range: 2.5–3) without subsequent GD reactivation in 96.2% of them. Twenty-two patients (10%) were hospitalized for COVID-19 and one (0.5%) required mechanical ventilation. Four (1.8%) died due to COVID-19 and one (0.5%) had long COVID-19 symptoms. Among patients in remission prior to SARS-CoV-2 infection, 22 (11.2%) experienced a GD relapse within 2.2 (range: 1.5–3.7) months from the diagnostic test. The relapse-free survival after COVID-19 was significantly shorter for patients with minimal change disease, pauci-immune glomerulonephritis, and focal segmental glomerulosclerosis. No difference was observed in the relapse-free survival post-COVID-19 based on the history of SARS-CoV-2 vaccination.

**Conclusions:**

SARS-CoV-2 infection appears to have a symptomatic but uncomplicated sequence in vaccinated patients with GDs, with a significant impact on the clinical course of GD, associated with an increased probability of relapse in certain histopathological types.

## Introduction

Since the emergence of severe acute respiratory syndrome coronavirus 2 (SARS-CoV-2) infection, over 700 million confirmed cases and over 6.9 million deaths have been reported worldwide (1, 2). Certain populations, including older patients and those with chronic conditions, appear to be at a higher risk for severe coronavirus disease 2019 (COVID-19) (3, 4). Glomerular diseases can result from inherited or acquired disorders and can manifest in a variety of ways, ranging in severity from asymptomatic urinary abnormalities to acute kidney injury, nephrotic syndrome, or rapidly progressive glomerulonephritis leading to end-stage kidney disease (ESKD). The diagnosis is generally made through a kidney biopsy revealing the underlying pathology. The severity of the disease is related to the nature and type of the immunologic process causing the disease and to the degree and extent of inflammation (5). Yet, patients with glomerular diseases (GDs) are known to be at a higher risk for severe infections due to an underlying immune dysfunction and renal impairment and as a consequence of long-term immunosuppressive therapy (6). Therefore, there is significant treatment-related morbidity in patients with GDs, associated with an increased incidence rate of severe infections (7). Treatment with high-dose oral corticosteroids was identified as the most important contributor being associated with up to seven-fold higher risk for infection occurrence (8). Additional risk factors for severe COVID-19 in these patients include a higher prevalence of hypertension and diminished kidney function (9, 10). Both GDs and COVID-19 are associated with a hypercoagulable state and thrombotic events causing high morbidity and mortality (11). Additionally, chronic immunosuppression, a frequent aspect of GD treatment, has been linked to a reduced response to SARS-CoV-2 vaccines and therefore might influence their effectiveness in preventing severe COVID-19. In this regard, patients with GDs have a weakened immunity due to disease activity and/or treatments than healthy subjects, and the third dose of the SARS-CoV-2 mRNA vaccine was shown to effectively increase the humoral immune response against SARS-CoV-2 and, to a lesser extent, the cellular response against SARS-CoV-2 (12). Furthermore, there is an added concern that COVID-19 may exacerbate GDs or cause acute kidney injury and increase the risk of kidney failure (13, 14). Relapses during the course of GDs are known to occur anyway, in different rates across histopathological types, i.e., 50%–75% in minimal change disease (MCD), one-third of cases in patients with membranous nephropathy (MN), and 30%–50% of cases in patients with pauci-immune glomerulonephritis. Taken all together, these patients are a particularly vulnerable group in the global COVID-19 pandemic, and information on how the illness affects their health position and GD activity is critical for their management.

We sought to investigate the clinical features of SARS-CoV-2 infection in patients with previously diagnosed GDs and evaluate the probability of associated GD reactivation in an actively monitored cohort of patients, throughout an observation period that spanned over the COVID-19 pandemic.

## Materials and methods

### Study design and inclusion criteria

This study was a multicenter retrospective observational study that included 10 centers from all over the Greek territory. Patients were eligible to be included in the study if they had biopsy-proven GD of autoimmune origin and positive polymerase chain reaction (PCR) test for SARS-CoV-2 infection after GD diagnosis. The included patients were studied retrospectively. Patients who received the GD diagnosis prior to SARS-CoV-2 vaccination or infection or had reached ESKD were excluded. Histopathological diagnosis of GD might be IgA nephropathy (IgAN), MN, MCD, primary focal segmental glomerulosclerosis (FSGS), lupus nephritis (LN), pauci-immune glomerulonephritis, IgA vasculitis, fibrillary glomerulonephritis, and C3 glomerulonephritis. Of these, podocytopathies are called the ones in which podocyte injury drives proteinuria and progressive kidney disease. They include MCD and FSGS. We recorded demographics; histopathological diagnoses; past medical history including history of hypertension, diabetes, or cardiovascular disease; immunosuppressive regimens and outcome of GDs; and information regarding vaccination against SARS-CoV-2, i.e., vaccine type and total number of doses administered. In terms of the SARS-CoV-2 infection, we recorded the reason for testing, i.e., symptoms versus exposure, as well as the type of symptoms (fever, myalgias, arthralgias, rhinitis, pharyngitis, anosmia, ageusia, fatigue, cough, dyspnea, respiratory failure). Information regarding hospitalization requirement; oxygen supplementation or mechanical ventilation; specific therapy for COVID-19 including glucocorticoids, remdesivir, or nirmatrelvir; and final outcome (full recovery, post-acute sequelae, death) was collected. The time interval between the diagnostic kidney biopsy and the diagnostic PCR test was captured. Patients with GDs and documented SARS-CoV-2 infection were managed with adjustments to immunosuppressants and other medication regimens at the clinician’s discretion (15, 16). The time period of observation was between the positive PCR test for COVID-19 to the last visit in the glomerular clinic.

Written informed consent from the participants or legal guardian/next of kin was not required to participate in this study in accordance with the national legislation and the institutional requirements.

### Characteristics of GD

Histopathological diagnosis and related immunosuppressive regimens were recorded (cyclophosphamide, glucocorticoids, mycophenolate mofetil, azathioprine, rituximab). Immunosuppressive therapy was considered from the time of GD diagnosis to the end of follow-up. The first outcome of the GD, i.e., after initial therapy, was recorded in all patients, which might be remission or active disease (patients with ESKD were excluded by study design). The patients’ GD activity status was assessed in every visit to the glomerular clinic before and after SARS-CoV-2 vaccination and infection up to the end of follow-up. Patients who experienced an alteration in activity status from remission to active disease were considered to have a GD relapse. In this regard, time to relapse was estimated counting from the time of SARS-CoV-2 infection to the documentation of GD relapse. Data collection prompted research coordinators to review medical records in each institution back to the time of SARS-CoV-2 infection in order to collect all related information. The included patients were required to have all information available regarding histopathological diagnosis, immunosuppressive treatment, and GD outcomes dating back to the diagnostic kidney biopsy. Definitions of GDs and related outcomes, including disease relapse, followed the ones provided by the Kidney Diseases Improving Global Outcomes (17). Activity status alteration might include change from active disease to remission or change from remission status to active disease, i.e., relapse. End-stage kidney disease was defined as the requirement of life-long maintenance dialysis. Acute dialysis was defined as the need for dialysis for less than 3 months. The estimated glomerular filtration rate (eGFR) was calculated using the Chronic Kidney Disease Epidemiology Collaboration (CKD-EPI) equation (18). Laboratory measurements with hematologic and renal indexes were performed throughout the follow-up time, including labs obtained during the closest visit to the glomerular clinic prior to infection (baseline laboratory measurements), the first visit after infection, and at the end of follow-up.

### Statistical analysis

The statistical analysis was performed using the R 4.0.4 packages “survival” (19) and “survminer” (20). A two-sided *p*-value of ≤0.05 determined statistical significance. We visually assessed the normality of continuous variable distributions by constructing and examining histograms. Variables found to be normally distributed were described using their mean and standard deviation, and the Student’s *t*-test was used for comparisons. The median and interquartile range were reported for variables that were not normally distributed, and the non-parametric Mann–Whitney *U* test was used for comparisons. When comparing categorical variables across different groups, we used the chi-square test or Fisher’s exact test if the assumptions of the chi-square test were not met. For the outcome of relapse-free survival, Kaplan–Meier curves were constructed and compared using the non-parametric log-rank test.

## Results

### Description of the study population

A total of 219 patients with a history of biopsy-proven GD and positive PCR test for SARS-CoV-2 were included in the study. All patients who received a GD diagnosis after SARS-CoV-2 vaccination or after infection were excluded (*n* = 64). The mean age of the study population was 44.4 ± 18.4 years and 105 (47.9%) were men. The major types of GDs were represented in this cohort as shown in **Table 1**. The vast majority of patients (88.1%) had a past medical history of hypertension, diabetes (10.9%), or cardiovascular disease (6.8%). Overall, 188 (85.8%) of the included patients had received at least one course of glucocorticoids as initial therapy for GD, while 81 (37%) had also received cyclophosphamide, 10 (4.6%) mycophenolate mofetil, 29 (13.2%) calcineurin inhibitors, and 15 (6.8%) rituximab. Most patients (89.5%) had achieved remission as a result of initial therapy. In total, 181 patients (82.6%) were vaccinated against SARS-CoV-2 with a median of 3 doses (range: 2.5–3). The BNT162b2 vaccine was the most commonly administered (95%), while 49.2% of the patients were on immunosuppressive therapy of any kind at vaccination. No symptom or sign of GD reactivation was observed in 181 (96.3%) of the patients, who were vaccinated against SARS-CoV-2 by the time of SARS-CoV-2 infection diagnosis. Seven patients experienced a GD relapse following vaccination (six patients with MCD and one patient with pauci-immune glomerulonephritis), which was treated accordingly, and all achieved remission prior to COVID-19 diagnosis, while 76 patients (34.7%) were on immunosuppressive therapy at the same time (17).

**Table 1:** Characteristics of patients with GDs who were diagnosed with SARS-CoV-2 infection.

Parameter N (%) or mean (±SD) or median [range]	SARS-CoV-2 infection (N=219)
Age at GD diagnosis (years)	44.4±18.4
Male gender	105 (47.9)
Histopathological diagnosis Pauci-immune glomerulonephritis Lupus nephritis IgA nephropathy Minimal change disease Membranous nephropathy Fibrillary glomerulonephritis C3 glomerulonephritis Focal segmental glomerulosclerosis IgA vasculitis Other	29 (13.2) 53 (24.2) 37 (16.9) 28 (12.8) 36 (16.4) 2 (0.9) 1 (0.5) 28 (12.8) 2 (0.9) 3 (1.4)
Past Medical History Hypertension Duration (years) Diabetes mellitus Duration (years) Cardiovascular disease Duration (years)	193 (88.1) 8.0 [5.0-13.1] 24 (10.9) 8.0 [4.0-11.2] 15 (6.8) 5.2 [2.1-8.2]
Initial therapy Cyclophosphamide Glucocorticoids Mycophenolate mofetil Calcineurin inhibitor Rituximab	81 (37.0) 188 (85.8) 10 (4.6) 29 (13.2) 15 (6.8)
Maintenance therapy Cyclophosphamide Glucocorticoids Mycophenolate mofetil Azathioprine Rituximab Rituximab ever	4 (1.8) 44 (20.0) 56 (25.6) 19 (8.7) 18 (8.2) 26 (11.9)
First outcome Remission Treatment resistant	N=209 187 (89.5) 22 (10.5)

### Clinical course of SARS-CoV-2 infection

The largest portion of patients (92.2%) underwent testing for SARS-CoV-2 infection due to symptoms (versus 7.8% after exposure). The most frequently reported symptom was fever (68.9%), among various other clinical manifestations (**Table 1**), while 10% of cases overall required admission to hospital for a median of 7 days (range: 5–10.8). Among the admitted patients, 5.5% required oxygen supplementation, but only one needed mechanical ventilation. Generally, patients (97.7%) experienced full recovery from SARS-CoV-2 infection, four patients died (1.8%) following complications of COVID-19, and one patient reported long COVID-19 sequela. In total, laboratory measurements including renal and hematologic indexes were similar before and after SARS-CoV-2 infection. Parameters related to COVID-19 and related outcomes in patients with GDs are shown in **Table 2**.

**Table 2:** Parameters related to the SARS-CoV-2 infection and related outcome in patients with GDs.

Parameter N (%) or median [range]	Patients with SARS-CoV-2
Vaccinated	181 (82.6)
Vaccine type BNT162b2 mRNA-1273 Janssen ChAdOx1 nCoV-19	172 (95.0) 7 (3.9) 1 (0.6) 1 (0.6)
Number of vaccine doses	3 [2.5-3]
Immunosuppression at vaccination	89 (49.2)
Immunosuppression at SARS-CoV-2 infection Cyclophosphamide Glucocorticoids Mycophenolate mofetil Calcineurin inhibitor Rituximab Azathioprine	76 (34.7) 0 (0.0) 35 (16.0) 30 (13.7) 18 (8.2) 6 (2.7) 6 (2.7)
Reason for testing Symptoms Exposure	202 (92.2) 17 (7.8)
Type of symptoms Fever Myalgias Arthralgias Rhinitis/Pharyngitis Anosmia/Ageusia Fatigue Cough Dyspnea Hospitalization Duration of hospitalization (days) Specific therapy Oxygen therapy Mechanical ventilation COVID-19-related thrombosis	151 (68.9) 9 (4.1) 5 (2.3) 52 (23.7) 7 (3.2) 32 (14.6) 72 (32.9) 6 (2.7) 22 (10.0) 7 [5-10.8] 42 (19.2) 12 (5.5) 1 (0.5) 3 (1.4)
COVID-19 outcome Recovery Post-acute sequelae Death	214 (97.7) 1 (0.5) 4 (1.8)

### SARS-CoV-2 infection and GD activity

**Figure 1:**
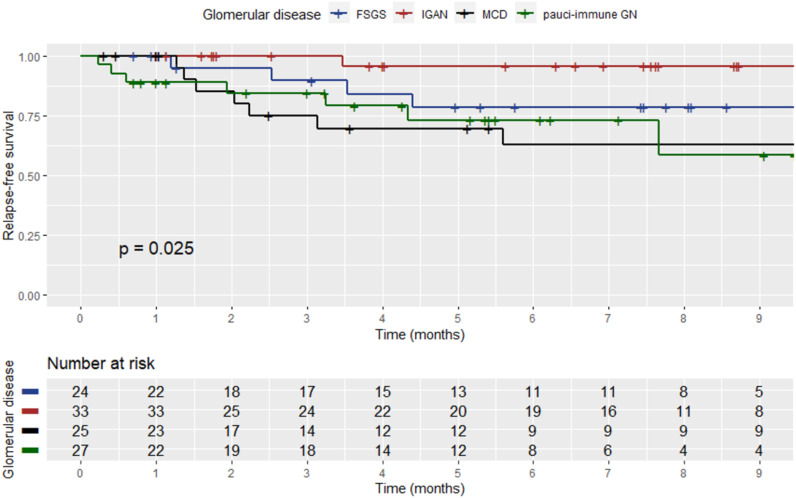
Kaplan–Meier curve showing the relapse-free survival post-SARS-CoV-2 infection among patients who experienced a relapse based on the histopathological type of GD.

During the visit prior to SARS-CoV-2 infection, 196 (89.5%) patients were in sustained remission for GD, while 23 (10.5%) had still active disease. In 22 patients (11.2%), an alteration of the GD activity status, i.e., from remission to active disease (i.e., GD relapse), was documented, within a median time of 2.2 months (range: 1.5–3.7) from the diagnostic SARS-CoV-2 test. Renal parameters and outcomes of GD post-SARS-CoV-2 infection are shown in **Table 3**. Among relapsers, there were six patients with pauci-immune glomerulonephritis (27.2%), two (9%) with renal lupus (one with lupus podocytopathy), eight (36.4%) with MCD, four (18.2%) with FSGS, one (4.5%) with IgAN, and one (4.5%) with MN. Notably, 14 (63.65%) of the relapsing patients had podocytopathies as primary disease and developed full-blown nephrotic syndrome upon relapse. The mean age of the patients who relapsed after COVID-19 was 49.2 years (±14.9), and there were 10 (45.5%) male patients. All relapsers had been vaccinated against SARS-CoV-2, and all except one were tested for COVID-19 due to symptoms (one due to exposure). Two of the relapsers required hospitalization for COVID-19. A comparison of baseline renal indexes between patients who relapsed after COVID-19 versus those who did not revealed that there was no difference in 24-h proteinuria, renal function, and microscopic hematuria (**Table 4**). However, at the time of GD relapse, patients with podocytopathies had significantly higher 24-h proteinuria versus the corresponding values at baseline (*p* = 0.0001), while serum creatinine did not differ. The relapse-free survival differed significantly by the histopathological type of GD (**Figure 1**) with MCD being the most frequently relapsing GD post-COVID-19. Yet, the relapse-free survival after COVID-19 was significantly shorter for patients with MCD, pauci-immune glomerulonephritis, and FSGS compared to patients with IgAN. Notably, seven (31.8%) patients who experienced a GD relapse post-COVID-19 had also documented GD reactivation after SARS-CoV-2 vaccination. There was no difference in the relapse-free survival post-COVID-19 based on the history of SARS-CoV-2 vaccination.

**Table 3:** Renal parameters and outcome of patients with GDs after SARS-CoV-2 infection.

Parameter N (%) or median [range]	
GD activity status at SARS-CoV-2 diagnosis Remission Active	196 (89.5) 23 (10.5)
Relapse post SARS-CoV-2 infection	22 (11.2)
Time to relapse from SARS-CoV-2 infection (months)	2.2 [1.5-3.7]
Renal indexes at baseline eGFR (ml/min/1.73 m^2^) Serum creatinine (mg/dl) 24-hour proteinuria (mg)	75 [52-99.5] 1.06 [0.80-1.50] 330.5 [126-895.8]
Hematuria (max RBC number/hpf)	2 [1-5]
Renal indexes at end follow up (all patients) eGFR (ml/min/1.73 m^2^) Serum creatinine (mg/dl) 24-hour proteinuria (mg) Hematuria (max RBC number/hpf) Follow-up time from SARS-CoV-2 infection (months) Total follow-up (from diagnostic kidney biopsy) (months)	71 [45-101] 1.10 [0.80-1.62] 409 [144-1206] 2 [1-5] 7.5 [2.4-10.8] 56.7 [30.1-108]

**Table 4:** Comparison of patients who experienced re-activation of the GD post COVID-19 and those who did not.

Parameter or median [range]	Patients with GD Relapse (N=22)	Patients without GD relapse (N=174)	p-value
Age (years)	47.5 [42.5-57.3]	43 [29-60]	0.177
Male gender	10 (45.5)	79 (45.4)	1
Histopathological diagnosis			
Pauci-immune glomerulonephritis Lupus nephritis IgA nephropathy Minimal change disease Membranous nephropathy Fibrillary glomerulonephritis C3 glomerulonephritis Focal segmental glomerulosclerosis IgA vasculitis Membranoproliferative glomerulonephritis	6 (27.3) 2 (9.1) 1 (4.5) 8 (36.4) 1 (4.5) 0 (0.0) 0 (0.0) 4 (18.2) 0 (0.0) 0 (0.0)	21 (12.1) 49 (28.2) 32 (18.4) 17 (9.8) 29 (16.7) 1 (0.6) 1 (0.6) 20 (11.5) 2 (1.1) 2 (1.1)	0.008
Immunosuppression at COVID-19 Glucocorticoids Mycophenolate mofetil Calcineurin inhibitor Rituximab Azathioprine	12 (54.5) 7 (31.8) 1 (4.5) 5 (22.7) 0 (0.0) 2 (9.1)	61 (35.1) 28 (16.1) 26 (14.9) 13 (7.5) 6 (3.4) 4 (2.3)	0.324 0.080 0.322 0.036 1 0.137
Hospitalization requirement	2 (9.1)	14 (8.0)	0.685
Duration of hospitalization (days)	5.5 [4.8-6.3]	6 [4.3-10]	0.688
Serum creatinine baseline (mg/dl)	0.99 [0.80-1.48]	1.00 [0.80-1.43]	0.859
24-hour proteinuria baseline (mg)	399 [160-650]	242 [103-656]	0.179
Hematuria baseline (max RBC number/hpf)	2 [2-5]	2 [1-4]	0.342

GD, glomerular disease; COVID-19, coronavirus disease 19; SARS-CoV-2, severe acute respiratory syndrome coronavirus 2.

## Discussion

This study investigated the clinical picture and outcome of COVID-19 in patients with GDs, as well as its impact, on the probability of disease reactivation. Most patients in this cohort underwent SARS-CoV-2 testing due to symptoms, while 34.7% were on immunosuppressive therapy at the time of diagnosis. The most frequent symptom of COVID-19 was fever, while 10% of the total (*n* = 219) required hospitalization for approximately 1 week. Nearly 98% of individuals experienced complete recovery from COVID-19 and 1.8% died due to related complications. Thus, the vast majority of patients in our cohort had symptomatic but uncomplicated COVID-19. Many patients with SARS-CoV-2 virus infection have various comorbidities. Their presence in the background of coronavirus tends to worsen the course of the disease and increase the risk of unfavorable outcomes (4). In this cohort of patients with biopsy-proven GDs, the clinical manifestations of COVID-19 were similar to the general population, in agreement with the findings previously reported (21). The United Kingdom’s ICHNT Renal COVID-19 group with systematic SARS-CoV-2 serology screening results in 493 patients with primary GD, vasculitis, and systemic lupus erythematosus prior to December 2020 reported that 19% of all cases required hospitalization and 7% resulted in death, pointing to substantial vulnerability of these patients. However, these rates were higher before the widespread use of SARS-CoV-2 vaccination (22). Generally, the clinical features of COVID-19 among patients with autoimmune disorders are variable but are not known to be different than patients without these underlying diseases. There are insufficient data from the literature to determine whether the type of autoimmune disease or the intensity of immunosuppressive therapy influences the clinical presentation of COVID-19. A systematic review investigating the impact of comorbidities found that patients with certain co-existing diseases are at a higher risk for severe courses of COVID-19 and unfavorable outcomes (23). These include heart diseases, immunosuppression, kidney diseases, metabolic syndrome, and hematologic diseases. Unmodified factors like older age and male sex also present an increased risk for severe events (23). Patients with GDs especially those on immunosuppressive therapy represent a population similar to kidney transplant recipients, who may also have some degree of renal impairment combined with a compromised immune system secondary to chronic immunosuppression. However, a meta-analysis, which compared the clinical outcome of COVID-19 in solid-organ transplant recipients, who typically receive long-term immunosuppression, to the one of the general population, found a higher risk of intensive care unit admission and occurrence of acute kidney injury but no difference in the 30-day mortality rate (24). Furthermore, we found that, among patients who had achieved long-term GD remission, 11.2% experienced documented disease reactivation, which occurred within 2.2 (range: 1.5–3.7) months after COVID-19. At first sight, the relapse rates after COVID-19 seem to be lower than the ones expected without this infection. However, it is of note that the time interval in which they have occurred is considerably shorter. Specifically, the relapse-free survival after COVID-19 was significantly shorter for patients with MCD, pauci-immune glomerulonephritis, and FSGS compared to patients with IgAN. Hence, SARS-CoV-2 infection appears to have a significant impact on the clinical course of GDs, associated with an increased probability of GD relapse in certain histopathological types. Nonetheless, COVID-19 induces a hyperimmune response in some patients which may hypothetically trigger a GD flare. Multiple mechanisms have been proposed for AKI, proteinuria, and hematuria in the setting of COVID-19. The cytokine storm induced may trigger common post-viral glomerular diseases such as IgAN, pauci-immune vasculitides, and anti-GBM that have also been described in COVID-19 patients. Also, direct viral invasion of glomerular structures is hypothesized to cause podocytopathy due to the affinity of the virus to angiotensin-converting enzymes. For decades, it has been observed that non-COVID viral infections are implicated in the relapse of nephrotic syndrome in pediatric patients and IgAN (25). While the mechanism of disease flare is not fully understood, it is thought to be due to a dysregulated immune response to infection. Patients may also be particularly vulnerable to a flare-up in the setting of withholding some of their immunosuppressive medication to allow a host immune response to the virus (26). For instance, MCD for 10%–15% of primary nephrotic syndrome cases in adults (27) while 30%–62% of patients experience a single relapse and up to 39% have frequent relapses (28, 29). Although the pathogenesis of MCD remains poorly understood, there is compelling evidence to suggest that it involves both immune system impairment and podocyte dysfunction. It is considered to involve intrinsic and extrinsic factors in a “long-chain” cascade reaction (30). It may be triggered by immunological stimuli, such as viral infection, immunization, allergens (31, 32), or chronic viral infection (33, 34). In children, a prospective two-winter study found that exacerbation and relapses of childhood nephrotic syndrome are temporally related to upper respiratory virus infections (35). The association of respiratory tract infections with the occurrence, relapse, and aggravation of nephrotic syndrome has been confirmed for over two decades (36). Moreover, human immunodeficiency virus (HIV) infection is a well-known cause of renal disease (37) and the most common cause of glomerular injury in these patients (38), triggered by the direct infection of glomerular epithelial cells and tubular epithelial cells by HIV (39). Likewise, the pathogenesis of pauci-immune vasculitides is thought to involve an interplay of environmental and epigenetic factors in genetically susceptible individuals (40). Seasonal and geographic variations in incidence provide insight into the potential role of environmental exposures in these diseases (41). Falk et al. and others found a seasonal variation in symptom onset, which was statistically higher than expected in winter and lower than expected in summer in patients with renal involvement (42–44). Moreover, Kemma et al. showed a seasonal influence on the risk of relapse at a rise of antineutrophil cytoplasmic antibodies (ANCA) in vasculitis patients with renal involvement (45). Specifically, ANCA rises, which occurred during the fall season, were more frequently followed by a relapse than ANCA rises occurring during other seasons (45). Although an ANCA rise might be directly pathogenic, this potential was considered unlikely, since fewer than half of the patients in whom an ANCA rise occurs will relapse within a year. Thus, a second hit is probably required (46, 47). In relation to this in a mouse model of MPO-ANCA, it has been demonstrated that proinflammatory stimuli of infectious origin and ANCA act synergistically to induce glomerulonephritis, which is in agreement with the hypothesis that patients at increased risk of a respiratory tract infection during the following month more frequently relapse and that antibiotic maintenance therapy did not protect against relapse after an ANCA rise (48).

With respect to the treatment effect, although it appears from this study that calcineurin inhibitor (CNI) therapy may be associated with an increased probability of relapse, this is an epiphenomenon and not a real effect, related to the primary disease and not the actual treatment, i.e., the CNI. Many patients with podocytopathies, i.e., MCD and FSGS (who frequently are on CNIs, especially after relapsing disease), experienced a relapse after COVID-19.

The limitations of this study pertain to its retrospective design and the fact that information regarding COVID-19 identification and clinical features relied on the review of medical records and patients’ reports in each institution. The lack of B-cell status in patients receiving rituximab and the vaccine immunity status in some of them are also limitations. However, associations with GD reactivation may be underrepresented due to potentially missing relapsing episodes related to the relatively short follow-up period after COVID-19. Our study design required participants to have had serum and urine laboratory tests performed before and after COVID-19, which precluded the inclusion of patients who did not have all tests available or may be biased toward those with more severe GDs who had more frequent monitoring.

In conclusion, according to our results, SARS-CoV-2 infection appears to have a symptomatic and sporadically complicated sequence in patients with GDs. It appears that COVID-19 may have a significant impact on the clinical course of GD, associated with an increased probability of relapse in certain histopathological types. However, some of these relapses may be related to the infection, whereas some of them may be coincidental. In favor of the relation to COVID-19 is the timing of relapse, i.e., occurring within a few weeks of the infection. Understanding the interactions between SARS-CoV-2 and GDs is key to the successful management of both COVID-19 and GD in the long term, which poses unique challenges, especially with regard to immunosuppression planning.

## Data Availability

The raw data supporting the conclusions of this article will be made available by the authors, without undue reservation.
